# Public Interest, Risk, Trust, and Personal Protective Equipment Purchase and Usage: Face Masks Amid the COVID-19 Pandemic

**DOI:** 10.3390/ijerph19095502

**Published:** 2022-05-01

**Authors:** Jie Feng, Jian Li, Wuyang Hu, Gucheng Li

**Affiliations:** 1 The School of Humanities and Social Sciences, Beijing Institute of Technology, 5 South Zhongguancun Street, Beijing 100811, China; jfeng65@wisc.edu; 2 The College of Economics and Management, Huazhong Agricultural University, Wuhan 430070, China; lgcabc@mail.hzau.edu.cn; 3 Department of Agricultural, Environmental, and Development Economics, The Ohio State University, Columbus, OH 43210, USA; hu.1851@osu.edu

**Keywords:** face mask, public interest, purchase, risk perception, trust, usage

## Abstract

This analysis considers public interest in COVID-19-related issues as well as individuals’ risk perception and trust in society in their demand for face masks during the pandemic. Through a national survey, we examine demand during both the outbreak and the recovery stage of the pandemic and differentiate demand into purchasing and usage. The examination allows us to observe the evolvement of demand over time and stockpiling. We find that public interest and risk perception had a more significant association with mask demand during the outbreak stage, and trust was more connected with mask demand during the recovery stage. While stocking was evident in both stages, consumers were much less price sensitive in the outbreak stage. Overall, the relationship between most factors and mask demand was smaller in the recovery stage. Our research is useful for policymakers to assess the creation and termination of temporary legislation to help manage the value chain of personal protective equipment during a major public health crisis.

## 1. Introduction

The coronavirus-19 (COVID-19) pandemic has led to dramatic losses of human lives worldwide and caused unprecedented challenges to public health, supply systems, and the way everybody works [1,2,3]. The world has witnessed unusual shortages and panicking stockpiling of food, hand cleanser, and personal protective equipment (PPE). Given the vast weakness in PPE preparedness across the world during this infrequent but catastrophic public health crisis [4,5], understanding the formation and transition of PPE shortage is crucial for being better prepared for possible future crises.

Shortages can come from multiple sources, including supply, demand, and distribution [6,7,8]. This paper targets the demand side for PPE and, in particular, face masks. Face masks are initially used to prevent the spread of bacteria and pathogens transmitted through the air [9]. In modern days, face masks are also seen in periods of severe air pollution. As a result, a study on face masks can generate practical implications for different wearing situations [10]. In this study, while we mainly focus on purchase, we also take usage into account since masks are storable, and purchases may not be equal to actual use in all given points of observation. Thus, we separate demand into two different aspects—purchase and usage. Moreover, we collect information on these two kinds of behavior during and after the pandemic through an online survey. To our knowledge, this is the first study that examines both the purchase and usage of face masks in response to the COVID-19 pandemic, which is also the first contribution of this study.

Building on the existing literature, we proceed by investigating factors that are related to PPE purchase and usage represented by face masks. Specifically, we examine the role of public interest, risk, and trust on mask demand. First, we construct a variable for public interest using web-scraped internet search counts. This variable is location-based and enables us to examine the impact of public interest on face mask demand. Second, we adopt a measurement of the risk aversion coefficient proposed by Barham et al. [11] to investigate the role risk preference plays in face mask demand. Third, trust in the overall society could reflect individuals’ confidence on whether they would also rely on others in the society to contribute to disease control. We thus follow the convention of the existing literature to use a Likert scale to measure individuals’ trust in society [12]. Putting these together, the second contribution of this study is to investigate the role of public interest, risk preference, and trust on the purchase and usage of face masks in a health crisis.

This current study applies to China since it has gone through two stages in terms of the evolution of COVID-19. We regard the first quarter of 2020 as the outbreak period when it was associated with a high incidence of confirmed cases, and most Chinese cities/towns experienced lockdowns. We treat the second quarter as the recovery period, during which confirmed cases decreased significantly. Since China is a large country with spatial differences in the spread of COVID-19, it allows us to further investigate spatial heterogeneity together with temporal effects. Because China has gone through both stages of the pandemic, experience learned in this process might also provide valuable implications for other countries.

The rest of the paper is organized as follows. Section 2 describes the survey and data. Section 3 discusses the econometric method. Section 4 presents empirical results on mask purchase and usage and further examines the role of public interest, risk, and trust. The conclusions and implications are summarized in Section 5.

## 2. Survey and Data

The data in this study come from a household survey using recall questions and experimental methods to obtain information on mask purchase and usage as well as that on risk, trust, and demographic factors. The survey was conducted using an online platform in China between August and September 2020. We adopted a proportional sampling scheme collecting respondents from each Chinese province based on population in that province but allowed within-province randomness. We used five rounds of focus groups in addition to two pilot surveys to improve the questionnaire design and data quality. After dropping incomplete observations, the survey collected 1054 representative respondents from 27 mainland provinces in China (In the final analysis, we eliminate the provinces with less than 10 observations. The number of observations from each province ranges from 14 to 109 in our sample).

### 2.1. Key Dependent Variables

The primary purpose of the survey is to collect information on face mask demand during and after the COVID-19 crisis. In the survey, we asked questions on mask purchase and usage in the first and second quarter of 2020 using the following question, “How many masks did your household purchase/use during the first/second quarter of 2020?”. This produces the dependent variables of mask purchase and usage in two periods varying across households.

### 2.2. Key Independent Variables

(a) Public interest. Unlike recent research that relies on a survey-based evaluation of public interest in COVID-19 [13], we construct a location-based objective measure using data on a public internet search on COVID-19 related keywords. Specifically, we first obtained the eight most frequently searched COVID-related keywords (the eight terms are “novel coronavirus”, “coronavirus lung disease”, “COVID-19”, “disease control”, “COVID-19 map”, “new data on COVID-19”, “live monitoring of coronavirus”, and “news update on COVID-19”) based on the largest internet search engine in China (www.baidu.com accessed on 1 February 2021), then web-scraped the total number of searches on these keywords in each city in the first and second quarter of 2020, respectively. Next, we added up the search number over the eight keywords in each city in our sample for each quarter and divided it by the city’s corresponding population. The population of each city was collected from the “2018 Annual Statistics of Major Cities” by the National Bureau of Statistics of China. As a result, our public interest variable varies across city and quarter.

(b) Risk aversion. The questionnaire also involves an experiment eliciting respondents’ risk preference based on the method in Barham et al. [11]. Building on the multiple-choice elicitation method [14], Barham et al. [11] develop an experiment that presents multiple numbers of choice scenarios for each respondent. In each scenario, the respondent was shown with a sure bet and a gamble, and the respondents would receive the corresponding payment based on these experiments and their choices. By varying the expected return of the gamble and recording the respondent’s choice in each scenario, one can measure the risk aversion coefficient of the respondent.

(c) Distrust. Our measurement of trust is based on a seven-point Likert scale from one (strongly agree) to seven (strongly disagree), taking the form, “In general, the majority of the society is trustworthy.” Ding et al. [12] used a similar trust scale in their measure of consumer preference for food products with controversial attributes, while Kim and Tandoc [15] used the scale in individuals’ decisions whether to wear a face mask. These past studies find that trust, in the government or in the general food system, helped to enhance compliance to advisories and tends to offset negative perceptions about the products being studied [16].

The survey also collected COVID-19-related variables and other demographic factors. COVID-19-related variables include the following: whether there are confirmed or highly suspected cases in the respondent’s social network; the total number of friends in the respondent’s social network; whether leaving the neighborhood community of their residence was restricted due to COVID-19. The survey also collected demographic variables including age, marital status, education, health status, family income, family size, and whether the family has children or the elderly.

### 2.3. Summary Statistics

(1) Changes in mask purchase and usage

**Figure 1:**
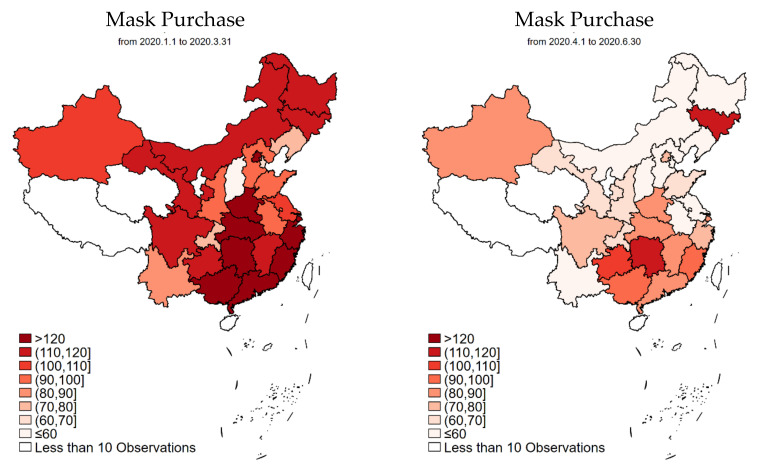
Face mask purchase during Period 1 and Period 2 in China at the provincial level.

**Figure 2:**
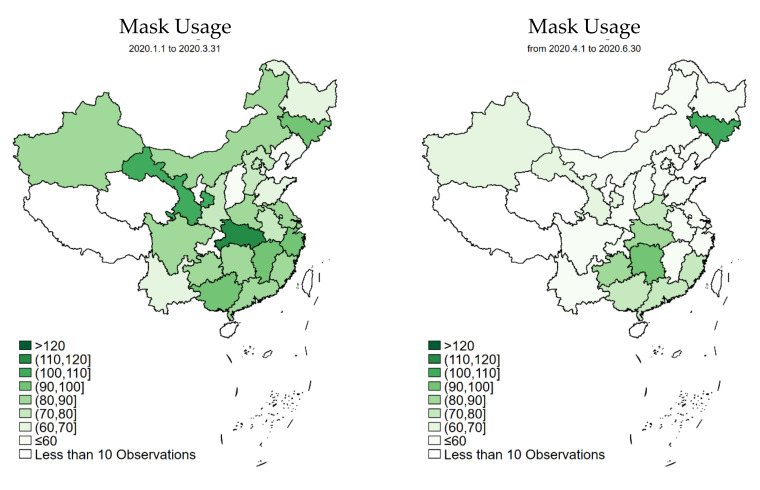
Face mask usage during Period 1 and Period 2 in China at the provincial level.

We regard the outbreak period from January 2020 to March 2020 as Period 1, and the recovery period from April 2020 to June 2020 as Period 2. These two periods also correspond to Q1 and Q2, respectively, of 2020. As a result, we use these timeframes interchangeably in our discussion. Table 1 reports the summary statistics of the main variables. The unprecedented COVID-19 outbreak in China triggered dramatic mask purchase and usage in Q1 2020, with the average per household (average 3.3 individuals per household) figure reaching 113.59 and 82.29 per quarter, respectively. Figure 1 further shows the top three provinces with the highest mask purchases: Hunan, Hubei, and Zhejiang, all of which experienced large numbers of COVID-19 cases. In contrast, during Q2 2020, though it still seems to be large, the per-household face mask purchase and usage reduced to 72.94 and 58.69, respectively. As shown in Figure 1 and Figure 2, at the province level, the new epicenter in Q2 2020 (i.e., Jilin, a northeast province) witnessed a high number of mask purchase and usage. While not shown in Table 1 and the figures, the data also indicate that mask purchase was higher in groups with higher income, larger family size, and families with children and elderly. Very similar results are found with mask usage.

Since individuals had an increasing demand for masks from the outbreak of the pandemic and they in general did not store masks prior to the pandemic, consumers intensively utilized masks in the two periods of our study. We find that mask usage accounted for 72.44% and 80.46% of the purchase in Period 1 and 2, respectively (We excluded respondents reporting the following: (1) unexpectedly high purchase and usage; i.e., those who purchased and/or used more than 500 masks over a three-month period; (2) unexpectedly high mask price, i.e., those with price higher than CNY 50/mask. This removed 1.65% of the data. Including these possible outliers does not change the nature of our findings). These numbers suggest two aspects: first, these fractions indicate the gap between expected usage (i.e., purchase) and actual usage; second, stocking was common. Expectedly, we find that the purchase and usage behavior identified in the outbreak period persisted in the recovery period (Q2 2020), though the difference between the number purchased and used was reduced. Since stored masks in Period 1 can be carried over to Period 2, we also re-calculated the ratio between the used and purchased in Period 2 under the assumption that all unused masks were carried over to Period 2. The adjusted ratio was 56.30% for Period 2. Regardless, our survey allows us to represent the spatial and temporal differences of face mask purchase and usage during the outbreak of COVID-19 and when it subdued to a large degree. Regression analysis to be presented in Section 4 below ties factors that may offer some explanation to what we observe here.

(2) Prices, public interest, risk, trust, COVID-related and other demographic factors

**Figure 3:**
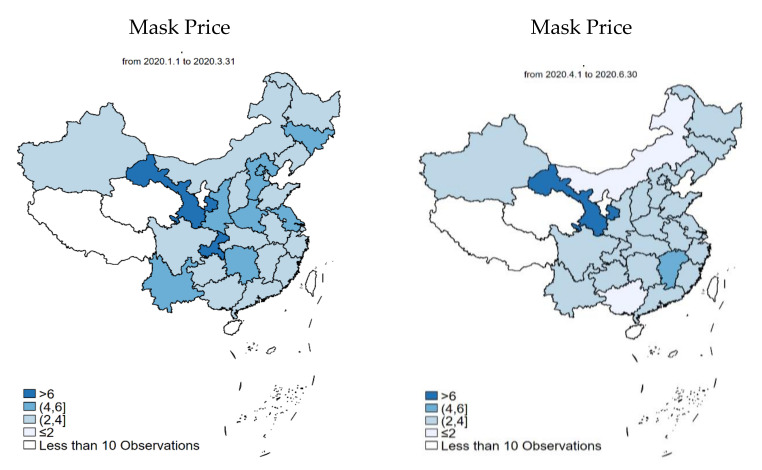
Average price of face masks during Period 1 and Period 2 in China at the provincial level.

Table 1 also shows the average price respondents reported that they have paid for face masks. In the outbreak period, it reached CNY 3.93 (with the exchange rate of about 6.5:1 between CNY and USD in January 2020, CNY 3.93/mask and 2.78/mask is approximately USD 0.60/mask and 0.43/mask, respectively) per mask, and dropped to CNY 2.78 in the recovery period. This may indicate the natural fluctuation in prices led by the supply demand relationship in the presence of a sudden and large-scale public health crisis. Figure 3 provides further information on spatial differences in mask prices at the provincial level. In Period 1, nearly half of the provinces experienced high mask prices with over CNY 2/mask, and the number largely reduced in Period 2. It is interesting to see that the epicenters (i.e., Hubei in Period 1 and Jilin in Period 2) did not experience unreasonably high prices, partially reflecting government interventions on price gauging.

The average number of COVID-19-related Internet searches representing public interest has a mean of 56.76 times per person (S.D. = 27.55) in Period 1 and 39.00 (S.D. = 19.27) in Period 2. This reduction in search is expected along with China’s recovery from the pandemic. The risk aversion coefficient shows a mean of 1.49, indicating that the average respondent is risk-averse [11]. This is consistent with the existing evidence that consumers tend to be risk-averse in general and is comparable to the numbers reported in Barham et al. [11]. The distrust variable has a mean value of 2.79, meaning respondents show a relatively high degree of overall trust in society. Table 1 also reports the summary statistics of other COVID-19-related variables and demographic factors. The sample was fairly representative of the Chinese population in terms of age (the national average is 38.8 years old) but had higher household income than the national average of CNY 32,189. Next, we proceed with the econometric analysis on factors associated with the changes in mask demand in response to COVID-19.

## 3. Econometric Analysis

### 3.1. Sample Selectivity Issue

The demand for face masks does not belong to essential day-to-day consumption, which requires constant refill now and then. Because of this nature, we can anticipate that there could be many cases of zeros in the number of mask purchase and usage absent of any public health incidents such as air pollution or a pandemic. Given COVID-19, we expect zero consumption would be more pronounced in the post-epidemic period, i.e., Period 2. From our survey data, we can see that 4.36% of the respondents did not buy any face masks, and 3.70% did not wear any masks in Period 1. These proportions raised to 31.40% and 31.12%, respectively, in Period 2.

In our analysis, if we assume the dependent variable, i.e., the purchase and usage of masks, is normally distributed, using the OLS method would lead to biased and inconsistent estimation results. Among a variety of options [17,18], we treat the dependent variables as left-censored at zero and utilize the Tobit model [19] to address the issue of truncation on mask consumption.

A second problem often tied with a traditional demand analysis is the issue of endogenous price, when the price is correlated with the error term due to unobserved characteristics. Fortunately, the face mask pricing mechanism during the pandemic period provides us the probability to eliminate the endogeneity problem by design. The State Administration for Market Regulation of China issued *the Notice on Resolutely Safeguarding the Price Order of the Market for Epidemic Preventative Products* on 25 January 2020, shortly after the pandemic outbreak (data source: China government website. Available at: http://www.gov.cn/zhengce/zhengceku/2020-01/31/content_5473364.htm accessed on 1 April 2022). The notice was to guide and urge market supervision departments at all levels to strengthen the supervision and inspection of the price of face masks and other personal preventative equipment and products. The rigid price ceiling enforced exogeneity on mask prices and can eliminate the endogeneity problem, similar to the regulated electricity prices in many countries, including China [20]. Furthermore, we also exclude observations with a reported price higher than CNY 50 (with an exchange rate of about 6.5:1 between CNY and USD in January 2020, CNY 50/mask is approximately USD 7.7/mask. We eliminated a total of six observations as a result of this operation) per mask to eliminate possible outliers (such as purchases made from informal or illegal sources). This adds additional support on price exogeneity. Finally, we added the quadratic form of the price term to capture the possible nonlinear price effect in our estimation.

### 3.2. Tobit Model

We assume that individual i, i=1,…,N at Period *t*, t=1,2, either purchases (j=1), or uses (j=2), $y_{ijt}$ masks. Since the number of masks is non-negative, we further assume $y_{ijt}^{*}$, the latent variable for the underlying purchased/used mask, to be left-censored at zero. The observed mask purchase and usage $y_{ijt}$ and the underlying variable $y_{ijt}^{*}$ have the following relationship represented by Equation (1).

(1) $$y_{ijt}=\left\{\begin{array}{c} y_{ijt}^{*},\;\mathrm{if}\;y_{ijt}^{*}>0\\ 0,\;\mathrm{if}\;y_{ijt}^{*}\leq 0 \end{array}\right.$$

Censoring of the observed mask consumption could be due to corner solution: mask price exceeds consumers’ willingness to pay, or some consumers have no household need for masks. The latent variable $y_{ijt}^{*}$, therefore, satisfies Equation (2).

(2) $$y_{ijt}^{*}=\mu^{\prime }X+\varepsilon_{ijt}$$

where vector $X=\left(x_{1},\;\ldots x_{n}\ldots ,x_{N}\right)$ contains all explanatory variables that could affect the latent mask demand, μ is a vector of associated coefficients to be estimated, and $\varepsilon_{jt}=\left(\varepsilon_{1jt},\ldots ,\varepsilon_{Njt}\right)$ is a random disturbance term assumed to follow a normal distribution $N\left(0,\sigma_{jt}^{2}\right)$.

The log-likelihood $lnL_{jt}$ is given as:

(3) $$lnL_{jt}=\overset{I}{\underset{i=1}{\sum }}\{\left(1-D_{ijt}\right)ln [1-\Phi \left(\frac{\mu^{\prime }X}{\sigma_{jt}}\right)]+D_{ijt}ln [\frac{1}{\sigma_{jt}}\phi \left(\frac{y_{ijt}-\mu^{\prime }X}{\sigma_{jt}}\right)]\}$$

where $D_{ijt}=\left\{\begin{array}{c} 1,\;\mathrm{if}\;y_{ijt}>0\\ 0,\;\mathrm{if}\;y_{ijt}=0 \end{array}\right.$ and $\Phi \left(\cdot \right)$ and $\phi \left(\cdot \right)$ are standard normal cumulative distribution function and probability density function, respectively. We estimate the purchase and usage of the masks in Period 1 and Period 2 separately to illustrate their evolvement and variation across periods.

The impact of each estimated coefficient in the Tobit model could be explained by its partial effects on the conditional or unconditional expectations of mask purchase/usage as shown in Equations (4) and (5), respectively:

(4) $$\frac{\partial E\left(y_{ijt}|y_{ijt}>0,\;X\right)}{\partial x_{n}}=\mu_{n}\left\{1-\frac{\phi \left(\frac{\mu^{\prime }X}{\sigma_{jt}}\right)}{\Phi \left(\frac{\mu^{\prime }X}{\sigma_{jt}}\right)} [\frac{\mu^{\prime }X}{\sigma_{ijt}}+\frac{\phi \left(\frac{\mu^{\prime }X}{\sigma_{jt}}\right)}{\Phi \left(\frac{\mu^{\prime }X}{\sigma_{jt}}\right)}]\right\}$$

(5) $$\frac{\partial E\left(y_{ijt}|\;X\right)}{\partial X_{n}}=\mu_{n}\Phi \left(\frac{\mu^{\prime }X}{\sigma_{jt}}\right).$$

To investigate the effect of prices, risk, distrust, and other variables on mask demand, we decompose $\mu^{\prime }X$ following:

(6) $$y_{ijt}^{*}=\alpha_{1jt}P_{i1t}+\alpha_{2jt}P_{i1t}^{2}+\beta_{1jt}\;Public\;interest_{it}+\beta_{2jt}\;Risk\;aversion_{i}+\gamma_{jt}Distrust_{i}+\delta_{jt}\prime Z_{ijt}+\varepsilon_{ijt}$$

where $\alpha_{1jt},\alpha_{2jt},\;\beta_{1jt},\;\beta_{2jt},\;\gamma_{jt},\;\mathrm{and}\;\delta_{jt}$ are unknown coefficients to be estimated, $P_{it}$ is the purchasing price at period t for individual i, $Public\;interest_{it}$ is the average number of searchers in the city where individual i was located during Period t, $Risk\;aversion_{i}$ is individual $i^{\prime }s$ risk aversion measure, $Distrust_{i}$ represents the level of the individual’s distrust in society, Z includes other control factors that could also have an impact on the demand for masks. These control variables include COVID-19-related factors, such as the following: the number of friends in the respondent’s social network; whether there were confirmed cases in the respondents’ social network; whether entry/exit of the community was restricted, as well as demographic factors such as household head age, marital status, education, health conditions, natural log of household income (CNY 1000), household size, and whether the household has any children or elderly.

In China, each province may have its additional specific regulations during the pandemic. The purchase and distribution of personal protective equipment are also regulated at the provincial level. Therefore, we also control the provincial level fixed effect. We remove data from provinces with less than 10 observations in our sample to avoid model convergence difficulty (we removed observations from the Chinese mainland provinces: Tibet, Ningxia, Qinghai, and Hainan for a total of 17 observations).

## 4. Results and Discussion

**Figure 4:**
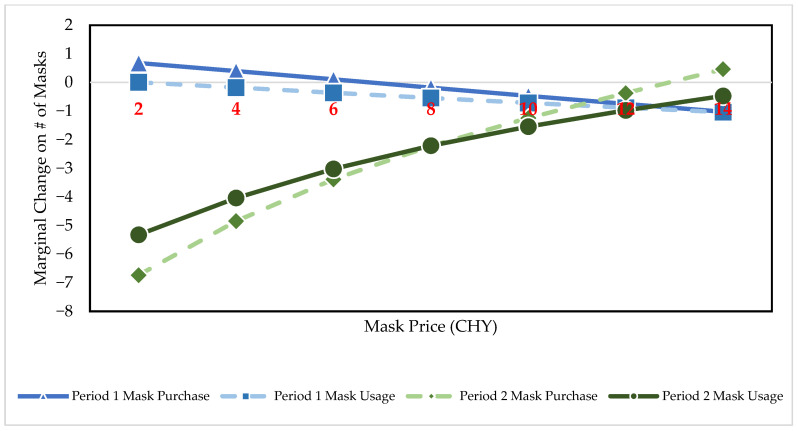
The unconditional marginal effects on number of purchased and used masks due to price change.

Table 2 illustrates the Tobit regression results on both mask purchase and usage in Period 1 and 2, respectively. The regression results show that price, public interest, risk, and distrust factors had different effects on purchase versus usage of face masks even within the same period. The F statistics and *p*-values reported in Table 2 show that our specifications are statistically significant overall. Our regression results also illustrate demand shifts between the two periods. Table 3 reports the unconditional marginal effect for all the respondents. Due to the nonlinear price effect, we further estimate the unconditional marginal effect of price, assuming it changes from CNY 2 to CNY 14. The price effects of mask purchase and usage in Period 1 and 2 are shown in Figure 4.

### 4.1. Baseline Results

We regard the outbreak period as our baseline. Therefore, we first focus on the first two columns in Table 2. The coefficients of Health and Age were negative and significant at the 10% and 5% levels, respectively, while *Public interest*, *Risk aversion*, *Social network*, and $Log\left(Income\right)$ had a positive and significant effect on mask purchase. The major differences between the first and second columns of Table 2 are the coefficients of Risk aversion, Distrust, Community restriction, Household size and Age. The usage of the face masks was more associated with *Distrust*, Community restriction and Household size, rather than Risk aversion, Poor Health and Age.

Since the coefficients in Table 2 are equal to their marginal effects only under the latent variable $y_{ijt}^{*}$, we utilize the estimated marginal effects to further interpret our findings. The magnitude of conditional marginal effects is greater than that of the unconditional ones, indicating the independent variables had a larger impact on the group who either purchased or used any face masks during the periods of study. Without loss of generality, we primarily focus on the unconditional marginal effects in Table 3 and Figure 4 (which specifically focuses on the marginal effect of price) in the following sections.

(1) The “disappeared” price effects

The first and second row in Table 2 show that price had an insignificant relationship with mask demand in the outbreak period. Figure 4 suggest that the marginal effect of price was only significantly different from zero when price was equal or larger than CNY 12 and CNY 10, respectively, for mask purchase and usage. This indicates that consumers might be insensitive to mask price change in the lower price range in Period 1. Considering the average mask price of CNY 3.93 in Period 1, consumers’ mask demand might not have been correlated strongly to price changes around the average level compared to other factors in the outbreak period.

(2) The role of Public Interest

The fifth row of Table 2 indicates that Public interest had positive connection with mask purchase and usage during the outbreak period at the 5% significance level. The unconditional marginal effect of Public Interest in Table 3 suggests that one more Internet research per household in the study period, on average, was correlated to an increase in both the mask purchase and usage by about 0.2.

Previous literature showed a positive relationship between *Public interest* on coronavirus or the number of searches on PPE and the spread of the pandemic [21,22,23]. Consequently, the relationship between mask purchase and usage and the city-specific measure of Public interest might also be due to the severity of the pandemic in a city or the average public interest in PPEs usage. Our research provides empirical evidence that Public interest could have significant correlation with mask consumption during the outbreak period.

(3) The role of Risk aversion

The fourth row of Table 2 illustrates that risk aversion had a significant and positive connection with mask purchase but had no connection with mask usage. Based on the fourth row in Table 3, respondents with one unit higher in the relative risk aversion index (i.e., more risk averse) would purchase 5.4 more masks on average.

Our findings are consistent with the existing literature that individuals who were more risk averse practiced more social distancing and mask-wearing [24]. In our case, since we separate the demand for masks into two actions, we can further observe that the relationship was more on buying instead of using. Since ultimately only masks used are truly “consumed”, our findings suggest that the increased demand observed at the store checkout associated with increased risk aversion may result in mask stocking.

(4) The role of Distrust

In contrast to risk aversion that could significantly increase mask demand, the fifth row in Table 2 shows that higher Distrust could be related to lower mask usage in Period 1. With each additional unit increase in social distrust level, our respondents would, on average, use 2.9 fewer masks unconditionally (see the fifth row in Table 3).

Our findings help demonstrate the relationship between social trust and individuals’ reactions towards the impact of the pandemic. Literature shows that residing in a trustful environment is more conducive to trusting others’ compliance to proactive social norms. For instance, Wu [25] presented that social and political trust was negatively correlated with confirmed COVID-19 cases. In our case, faith in society might have led people to strictly execute the mask orders, thus increasing the need to use masks.

(5) Other COVID-19-related and demographic factors

Among social interaction variables, Social network had a significant and positive connection with both mask purchase and usage at the 1% and 5% levels, respectively, while Community restriction was only significantly related to mask usage. The estimated unconditional marginal effect of Social network in the sixth row in Table 3 indicates that each additional individual in a person’s social friend network was associated with 0.06 and 0.02 more in mask purchase and usage, respectively. Individuals with a larger social network might hear more updates about the pandemic, which may, in turn, lead to the availability heuristic (availability heuristic, also known as availability bias, refers to the cognitive heuristic through which the frequency of an event is judged by the instance bringing attention to an individual (Oxford Reference, https://www.oxfordreference.com/view/10.1093/oi/authority.20110803095436724 accessed on 1 April 2022) [26,27]). Consequently, they might wear or buy more masks than those with a smaller friend circle.

Community restriction was significantly related to the increase in mask usage by 9.5 but uncorrelated to purchase, as shown in the eighth row in Table 3. Entry/exit restrictions of the local community might often be accompanied by social regulations on mandatory mask-wearing in public spaces, and the restrictions might also be an indicator of the severity of the local outbreak. Consequently, individuals may tend to wear masks more often in areas with stricter entry/exist restrictions.

During the COVID-19 outbreak, socio-demographic factors such as education, marital status, and composition of family members did not affect the demand for masks, as shown in Table 2. On the other hand, respondents who were one year older would purchase 0.6 fewer masks on average, and individuals with one level higher in self-assessed Poor Health, i.e., with worse health conditions, would decrease their purchase and usage of masks by 7.6 and 4.3 on average, respectively (based on Table 1, sample average self-assessed health status is 3.28 (between neutral and poor), a one-level increase would indicate a health status between good and neutral). Older and self-reported unhealthy people might have a lower tolerance for health risks. They might expect themselves to stay at home more often such that they would purchase fewer masks, while their actual mask usage was not strongly related to their health status since when they had to leave home, they were subject to the same mask-wearing restrictions as the others. Meanwhile, respondents on average utilized 3.3 more masks in this period with one more family member, but the unconditional marginal effect was insignificant with mask purchase.

The coefficient of $Log\left(income\right)$ was positive and significant for both mask purchase and usage in Table 2, indicating that masks might be treated as a normal good during the pandemic with a positive income effect. Families with higher household income bought and used significantly more masks. With a 100% increase in family income, based on the marginal effect in Table 3, individuals would, on average, purchase 6.1 more masks and use 4.1 more masks.

### 4.2. Temporal Effects

The Tobit estimation results of Period 2, listed in the third and fourth columns of Table 2, illustrate the relationship between the same explanatory variables as in Period 1 with mask purchase and usage in Period 2, i.e., the recovery period. We first briefly summarize the overall results and move on to explain individual effects in more detail afterward.

Both P and $P^{2}$ were significant at the 1% level. The coefficient of Risk aversion, again, was only related to purchasing rather than usage. Trust was negative and significant for both mask purchase and usage at 10% and 1% level. For the social interaction variables, one of the major differences between the outbreak and recovery period is that Community restriction had a significant and positive connection with mask purchase at the 1% level in Period 2. The effects of socio-demographical variables also changed between the two periods. Married was now significant (marginally) for mask usage; Age was significant for both purchase and usage; while Poor Health and $Log\left(income\right)$ were no longer significant, and Household size was now significant for both decisions. We further focus on the third and fourth columns of Table 3 to explain these variables’ unconditional marginal effects in detail.

(1) The recovered price effects

One of the most distinctive characteristics of Period 2 is that the marginal effect of price was significant. As shown in the second column in Table 3, the marginal effect of P was negative and significant, and that of $P^{2}$ was positive and significant, both at the 1% level, showing a convex demand function [28,29,30]. Again, due to nonlinearity, we estimate the unconditional marginal effect of price, and illustrate them in Figure 4. A noteworthy finding that differed from Period 1 is that consumers became sensitive to price fluctuations as low as CNY 2 in Period 2.

Figure 4 shows that the marginal effect of price on mask purchase and usage was negative before the price reached CNY 10 and CNY 12 for purchase and usage decisions, respectively. In addition, the effect size diminished gradually with the increase in price. For instance, individuals would reduce their purchase by 6.7 with CNY 1 increase in price from CNY 2, while the same effect was reduced to 1.2 at CNY 10. Similarly, as in purchasing, consumers would use 5.3 fewer masks with CNY 1 increase at CNY 2, while the effect decreased to 1.5 at CNY 10. Furthermore, individuals did not appear to change their purchasing or usage behavior substantially at higher prices.

We believe these findings are consistent with our expectations. By the end of March 2020, the daily newly reported cases in China dropped to around ten (Data source: National Health Commission of China. Available at: http://www.nhc.gov.cn/xcs/yqtb/202103/1d619d17c6e14850a02e3e571df54b31.shtml accessed on 1 April 2022). The average price of masks in the second stage also dropped to CNY 2.78 per mask compared to CNY 3.91 in the first stage. Consequently, consumers might have regained their price sensitivity, so the marginal effect of price was negative and significant for most price levels considered in Period 2, regardless of purchase or usage. We conclude that compared with the first stage, individuals’ purchase and usage of masks might have been realigned with regular behavior associated with a normal good.

(2) The “disappeared” effect of Public interest

Among factors related to COVID-19, the coefficient and associated unconditional marginal effect of Public interest shown in the third row of Table 2 and Table 3 was no longer significant, which might indicate a relief of the public concern or fear over the unknown pandemic and possible supply shortage of PPE including face masks.

(3) A decreasing marginal effect of Risk aversion

Risk aversion played a similar role as in Period 1. The coefficient as well as unconditional marginal effect of Risk aversion on mask purchase, shown in the fourth row of Table 2 and Table 3, respectively, was still positive and significant for mask purchase at the 5% level in Period 2. However, the magnitude decreased to a 3.2 reduction in mask purchase per 1 point increase in risk aversion. This finding is consistent with previous literature on expected utility theory that risk aversion has a more prominent effect in situations where the probability of risk or the value of the potential loss is higher [31].

(4) An increasing marginal effect of Distrust

Trust in the second period of the pandemic had a slightly different impact compared to the first period. It was significantly associated with mask purchase and usage at the 10% and 1% significance levels, respectively. One level increase in Distrust was associated with 2.5 fewer mask purchases and 3.6 fewer masks used based on the fifth row in Table 3, and both were larger than the effects in Period 1. This indicates that Distrust might have played a more significant role in the post-outbreak period.

(5) Other COVID-related and demographic factors

Other factors related to COVID-19 and consumer demographic characteristics had a similar effect as in Period 1. Social network had the same qualitative effect, while the effect for purchase and usage also became similar, as shown in the sixth row of Table 3. The unconditional marginal effect of 100 more friends in an individual’s social network was associated with 4.0 more mask purchases and 2.8 more masks used. Community restriction became one of the major factors that was strongly positively connected with both mask purchase and usage. Based on the eighth row of Table 3, the local ban would be associated with mask purchase and usage by 19.9 and 15.3, respectively. The possible reason might also be that Community restriction was a direct indicator for the severity of local outbreak. In Period 2, while most other regions of China were free of new infections, community restriction would occur only in localities that were facing high risks of outbreaks. As a result, mask purchase and usage were expected to rise along with areas identified as high-risk areas.

The ninth to the fifteenth row of Table 2 and Table 3 display the coefficient estimates and marginal effects of demographic factors. Different from Period 1, Age on both mask purchase and usage were negative and significant at 5% and 10% levels, respectively. Married respondents were associated with using 7.7 fewer masks during Period 2. Like in Period 1, Education  and Children_elderly were also insignificant in Period 2. However, Poor Health and $Log\left(income\right)$ became statistically insignificant for both mask purchase and usage. The unconditional marginal effect of Household size was significant for both mask purchase and usage. On average, families with one additional member would purchase 5.6 more masks and use 4.0 more during Period 2. These findings once again might reflect the change in mask purchase and usage along with the society returning to normal after the initial outbreak of COVID-19.

## 5. Conclusions

This paper presents an empirical analysis of the pattern and factors contributing to personal protective equipment consumption in response to a major public health crisis. With a survey containing 1054 Chinese consumers reporting their facial mask purchasing and usage information during both the outbreak and recovery periods of COVID-19, we observe several key results with related economic implications.

First, by using national data and including multiple periods, we represent temporal differences in mask consumption while controlling for spatial differences across the country. We further differentiate consumption into purchase and usage, allowing us to reflect on stockpiling. We find that COVID-19, as expected, triggered a large amount of mask purchases and usage in the first quarter of 2020, especially in provinces with higher confirmed cases. Purchase and usage persisted in the recovery period, though both amounts were reduced, and factors associated with both types of behavior changed over the two periods. In addition, factors associated with purchasing and usage were also different.

Second, we find that public interest in knowing about the pandemic, risk, and distrust factors all were strongly related to household mask purchase and usage. More public interest and higher risk aversion were associated with more mask consumption, but the marginal effects were stronger for purchase than for usage, especially with the risk aversion variable. Furthermore, these effects were less pronounced in the recovery period. Distrust, on the other hand, mainly affected mask usage rather than purchase in both periods. Finally, we find that price was not a significant factor determining mask consumption in the outbreak period, suggesting that during the peak of the pandemic, consumers might be more concerned about having access to masks instead of the price they pay for the masks. We also uncover evidence of nonlinear price effect in mask purchase and usage in the recovery stage, indicating masks became more of a regular consumer product.

This study has several policy implications. First, as the pandemic affects PPE supply and demand, we show that differentiating stages of the pandemic and different types of consumption can be essential. Since households can easily store face masks and our result does show some evidence of stockpiling, appropriate policy should be in place to discourage unnecessary hoarding, especially in the pandemic outbreak stage. As much as rationing is undesirable for sellers carrying PPE products, such a tool may be proven necessary in the event of a pandemic. In addition, since consumers were not sensitive to prices in the outbreak period, anti-price-gouging does seem to have the potential to avoid market distortion and protect consumers, especially those with low-income. Since both purchase and usage of masks dropped in the recovery period immediately following the outbreak period, there does not appear a need for a long-term policy to regulate the market. Governments can consider ending emergency market regulations promptly following the conclusion of a public health shock to avoid long-term adverse market impacts.

Second, since public interest, risk aversion, trust in the society, and social network all had significant connection with mask purchase or usage, public policy affecting the formation and evolvement of these factors may also need to be assessed carefully to incorporate the indirect effect of the policy on PPE consumption through the induced changes of these individual characteristics. Some of these changes may take time, but the development of modern communication technology (e.g., publishing official information using main-stream social media) may shorten that time and reduce the possibility of stockpiling. For instance, during the outbreak period, information on pandemic-related or quarantine policies disseminated over the internet generated public interest, reshaped individuals’ social networks and restored trust in each other, all in turn could affect PPE purchase and usage.

Finally, as a caveat, the data used in this study are from a recall survey and they may suffer from recall bias. In our survey, in a section not specifically related to only masks, we asked respondents how well they could recollect their household purchasing record during and after the initial outbreak period. In our focus groups in addition to the two pilot surveys, respondents indicated they had little difficulty recalling their purchases. We attribute this to the heightened attention individuals had been giving to the pandemic and household purchases directly related to the pandemic. Nevertheless, future work may combine recall survey data and revealed market data to offer additional insights.

## Figures and Tables

**Table 1:** Definition and summary statistics of main variables.

Variable	Description	Mean	S.D.
$Purchase_{1}$	mask purchase in Period 1	113.59	105.87
$Purchase_{2}$	mask purchase in Period 2	72.94	89.76
$Usage_{1}$	mask usage in Period 1	82.29	78.78
$Usage_{2}$	mask usage in Period 2	58.69	76.29
$P_{1}$	mask price in Period 1	3.93	3.94
$P_{1}^{2}$	mask price square in Period 1	31.01	93.57
$P_{2}$	mask price in Period 2	2.78	2.79
$P_{2}^{2}$	mask price square in Period 2	15.50	74.32
$Public\;interest_{1}$	average number of searches per person in Period 1 (per 1000)	56.76	27.55
$Public\;interest_{2}$	average number of searches per person in Period 2 (per 1000)	39.00	19.27
*Risk aversion*	risk aversion coefficient based on Barham et al. [11]	1.49	1.27
*Distrust*	overall distrust in society *	2.79	1.25
*Social network*	# of friends in social network (e.g., WeChat)	227.03	189.30
*Confirmed case*	dummy on whether there are confirmed/suspected cases in respondent’s social network in either period	0.08	0.28
$Community\;restriction_{1}$	dummy on whether going out of community was restricted in Period 1	0.52	0.50
$Community\;restriction_{2}$	dummy on whether going out of community was restricted in Period 2	0.16	0.37
*Age*	age of respondent	33.91	7.42
*Married*	married = 1; 0 otherwise	0.22	0.41
*Education*	highest completed level of education **	5.84	0.63
*Poor Health*	self-stated poor health status ***	1.95	0.71
*Log(Income)*	natural log of household pre-tax monthly income (CNY 1000)	20.33	13.24
*Household size*	number of members in household	3.28	1.04
*Children_elderly*	whether the household has children or elderly	0.76	0.42

* We ask subjects to rate their level of agreement on the following statement: “in general, the majority of the society is trustworthy”. 1 represents strongly agree; 2, agree; 3, to some extent agree; 4, neutral; 5, to some extent disagree; 6, disagree; 7, strongly disagree. ** Education level: 1, primary school drop-outs; 2, primary school; 3, middle school; 4, high school or equivalent; 5, some college; 6, university; and 7, master and above. *** We ask subjects to rate their usual state of health on the following statement: “what is your usual state of health?” 1 represents very good; 2, good; 3; neutral; 4, poor; and 5 very poor.

**Table 2:** Tobit regression results for Period 1 and Period 2.

Independent Variables	Period 1		Period 2	
	Mask Purchase	Mask Usage	Mask Purchase	Mask Usage
P	1.481	0.293	−16.376 ***	−12.649 ***
	(1.902)	(1.430)	(4.214)	(2.641)
$P^{2}$	−0.110	−0.071	0.635 ***	0.396 ***
	(0.070)	(0.051)	(0.230)	(0.125)
*Public interest*	0.362 *	0.339 **	0.042	0.365
	(0.213)	(0.148)	(0.294)	(0.261)
*Risk aversion*	8.202 ***	−0.546	6.452 **	3.722
	(2.737)	(1.945)	(2.929)	(2.436)
*Distrust*	−1.692	−4.451 **	−5.289*	−7.912 ***
	(2.722)	(2.060)	(3.077)	(2.699)
*Social network*	0.095 ***	0.027 **	0.083 ***	0.059 ***
	(0.020)	(0.013)	(0.020)	(0.017)
*Confirmed case*	−10.107	6.194	2.098	13.629
	(11.768)	(10.338)	(13.384)	(12.366)
*Community restriction*	6.194	14.752 ***	39.771 ***	32.979 ***
	(7.068)	(4.889)	(10.027)	(8.162)
*Age*	−0.886 *	−0.234	−1.367 **	−0.984 *
	(0.532)	(0.384)	(0.644)	(0.557)
*Married*	−11.510	−4.476	−15.282	−16.492 *
	(10.015)	(6.715)	(10.896)	(9.200)
*Education*	−4.765	−2.328	10.673 *	7.722
	(6.026)	(4.292)	(6.281)	(5.210)
*Poor Health*	−11.621 **	−6.648 *	6.362	7.447
	(4.993)	(3.662)	(5.627)	(4.850)
*Log*(*Income*)	9.419 **	6.489 **	2.039	0.325
	(3.880)	(3.042)	(4.794)	(3.679)
*Household size*	1.684	5.140*	11.868 ***	8.656 **
	(3.644)	(2.966)	(3.941)	(3.459)
*Children_elderly*	6.843	5.284	−3.989	6.011
	(9.772)	(6.597)	(11.507)	(9.411)
*Provincial Fixed Effect*	Yes	Yes	Yes	Yes
Constant	100.279 *	39.508	5.531	−16.584
	(55.210)	(39.150)	(59.349)	(52.858)
Sigma	102.631 ***	77.130 ***	110.112 ***	92.841 ***
	(3.464)	(2.801)	(4.146)	(3.822)
Log Likelihood	−6145.298	−5891.611	−4728.936	−4680.781
F Statistics	3.644	3.115	194.788	4.149
(*p* Value)	0.0000	0.0000	0.0000	0.0000
N	1054	1054	1054	1054

Note: Robust standard errors are listed in parentheses. Asterisks indicate the significance level: * at the 10 percent level, ** at the 5 percent level, and *** at the 1 percent level.

**Table 3:** Estimated unconditional marginal effects for purchase and usage for Period 1 and Period 2.

Independent Variables	Period 1		Period 2	
	Mask Purchase	Mask Usage	Mask Purchase	Mask Usage
P	0.966	0.189	−7.770 ***	−5.913 ***
	(1.239)	(0.919)	(2.002)	(1.232)
$P^{2}$	−0.072	−0.046	0.301 ***	0.185 ***
	(0.046)	(0.032)	(0.109)	(0.058)
*Public interest*	0.236 *	0.218 **	0.020	0.171
	(0.139)	(0.095)	(0.140)	(0.122)
*Risk aversion*	5.351 ***	−0.351	3.061 **	1.740
	(1.790)	(1.251)	(1.391)	(1.139)
*Distrust*	−1.104	−2.862 **	−2.510*	−3.699 ***
	(1.776)	(1.324)	(1.462)	(1.263)
*Social network*	0.062 ***	0.018 **	0.040 ***	0.028 ***
	(0.013)	(0.008)	(0.010)	(0.008)
*Confirmed case*	−6.594	3.982	0.996	6.371
	(7.677)	(6.643)	(6.349)	(5.769)
*Community restriction*	4.041	9.485 ***	18.871 ***	15.417 ***
	(4.611)	(3.137)	(4.767)	(3.818)
*Age*	−0.578 *	−0.150	−0.649 **	−0.460 *
	(0.347)	(0.247)	(0.305)	(0.259)
*Married*	−7.510	−2.878	−7.251	−7.710 *
	(6.533)	(4.320)	(5.163)	(4.300)
*Education*	−3.109	−1.497	5.064 *	3.610
	(3.927)	(2.756)	(2.980)	(2.438)
*Poor Health*	−7.582 **	−4.274 *	3.019	3.481
	(3.257)	(2.358)	(2.672)	(2.266)
*Log(Income)*	6.145 **	4.172 **	0.968	0.152
	(2.526)	(1.950)	(2.275)	(1.720)
*Household size*	1.099	3.305 *	5.631 ***	4.046 **
	(2.378)	(1.905)	(1.871)	(1.615)
*Children_elderly*	4.465	3.398	−1.893	2.810
	(6.377)	(4.242)	(5.458)	(4.404)
N	1054	1054	1054	1054

Note: Robust standard errors are listed in parentheses. Asterisks indicate the significance level: * at the 10 percent level, ** at the 5 percent level, and *** at the 1 percent level.

## Data Availability

The data presented in this study are available on request from the corresponding author. The data are not publicly available due to the privacy restrictions.
